# Electrochemotherapy treatment safety under parallel needle deflection

**DOI:** 10.1038/s41598-022-06747-x

**Published:** 2022-02-17

**Authors:** Daniella L. L. S. Andrade, Raul Guedert, Guilherme B. Pintarelli, Marcelo M. M. Rangel, Krishna D. Oliveira, Priscila G. Quadros, Daniela O. H. Suzuki

**Affiliations:** 1 Institute of Biomedical Engineering, Federal University of Santa Catarina, Florianópolis, 88040-900 Brazil; 2 Oncology Veterinary, VetCâncer, São Paulo, 04523-013 Brazil

**Keywords:** Targeted therapies, Computational models, Biomedical engineering

## Abstract

Electrochemotherapy is a selective electrical-based cancer treatment. A thriving treatment depends on the local electric field generated by pairs of electrodes. Electrode damage as deflection can directly affect this treatment pillar, the distribution of the electric field. Mechanical deformations such as tip misshaping and needle deflection are reported with needle electrode reusing in veterinary electrochemotherapy. We performed in vitro and in silico experiments to evaluate potential problems with ESOPE type II electrode deflection and potential treatment pitfalls. We also investigated the extent to which the electric currents of the electroporation model can describe deflection failure by comparing in vitro with the in silico model of potato tuber (*Solanum tuberosum*). The in silico model was also performed with the tumor electroporation model, which is more conductive than the vegetal model. We do not recommend using deflected electrodes. We have found that a deflection of ± 2 mm is unsafe for treatment. Inward deflection can cause dangerous electrical current levels when treating a tumor and cannot be described with the in silico vegetal model. Outward deflection can cause blind spots in the electric field.

## Introduction

Electrochemotherapy (ECT) is an anti-cancer procedure that uses electroporation (EP) to improve traditional chemotherapeutic treatments^1^. EP is a physical phenomenon of cell permeability-increasing by exposition to high-intensity pulsed electric fields^2^. Studies from science, technology, engineering, and mathematics play a key role in EP-based treatment safety improvement. Investigations include analysis of electric field diffraction due to adjacent tumor structures, such as vascular^3,4^, eyelid-periocular skin^5^, and auxiliary methods to maximize the tumor treatment^6,7^. The pulsed electric fields are generated by dedicated hardware and delivered to the tissue using arrangements of plate or needle electrodes. The European Standard Operating Procedures on Electrochemotherapy (ESOPE)—first released in 2006^8^ and updated in 2018^9^—standardizes four main types of electrodes for ECT procedures.

**Figure 1:**
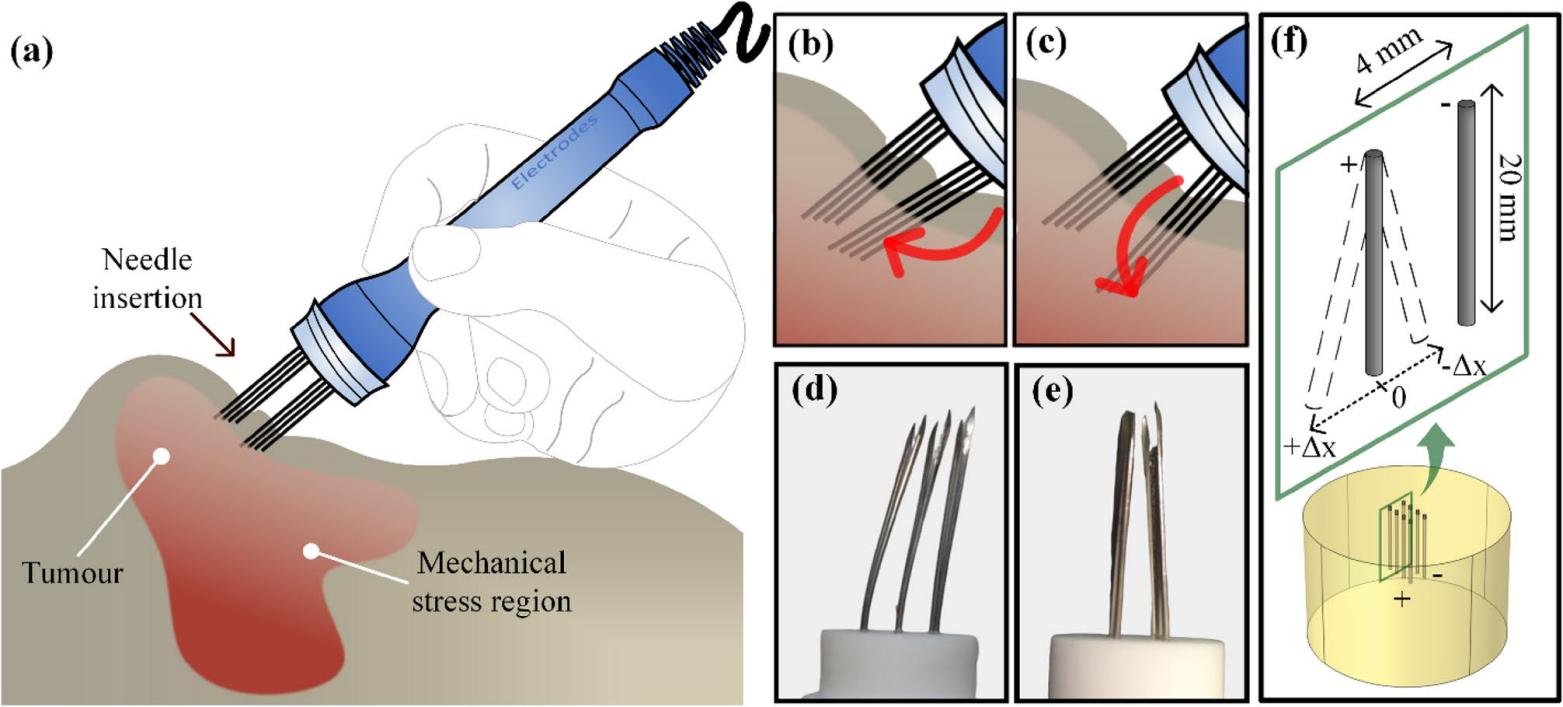
Illustration of electrode deflection in a mechanical stress region due to needle insertion. **(a)** The electrode insertion in stiff tissues may cause **(b)** an inward deflection or **(c)** an outward deflection. Two possible directions of needle deflection are shown. Illustrations were created in Microsoft VISIO Professional 2019 v.2111 (Microsoft Corporation, Washington, USA; https://www.microsoft.com/en-us/microsoft-365/visio/flowchart-software) **(d,e)** are two cases of electrode deflection returned to electrode manufacture. **(f)** Numerical simulation geometry showing the ESOPE Type II electrode inserted into the tissue with standard configuration. The geometry was generated by COMSOL Multiphysics v.5.1 (COMSOL Inc., Stockholm, Sweden; https://www.comsol.com/comsol-multiphysics). The highlight depicts the directions for the anode needles deflection studied in this work.

Although ESOPE does not report instructions about electrode refurbishment, it is commonly practiced in veterinary procedures reusing the electrode after autoclave sterilization or during multiple perforations in the same treatment^10,11^. Solid tumors increase the collagen fiber density in the extracellular matrix and adjust tissue stiffness^12^. Reuse of electrode produces mechanical deformations due to mechanical stress, especially during the needle electrode penetration on tumor tissue^12^, as illustrated in Fig. 1a–c. Needle tip misshaping—decreasing its sharpness—and needle body deflection are the most common reported deformations in needle electrodes (see Fig. 1d,e)^13–16^. Needle tip misshaping introduces immediate perforation risk to the safety of procedures. Needle deflection is a greater concern when analyzed in an electrical scope as field distribution will differ from the engineering design.

The success of ECT procedure depends on the electric field distribution on the tissue^3,17^. To guarantee that the tissue is electroporated, it must receive a minimum electric field intensity called reversible EP threshold (E_RE_)^18^. The electric field is a physical quantity related to the applied potential and the distance between the charges, in the case of ECT electrodes, to the oppositely charged needles. Under needle deflection, the entire electric field distribution on the tissue may change and compromise treatment safety and success. Inward deflection (when opposite charge needles become closer than usual) can introduce higher electric fields, higher electric current, and more undesired ablation in greater tissue extension by irreversible EP (IRE). IRE occurs when the electric field intensity surpasses the irreversible EP threshold (E_IRE_), directly killing the cell by osmotic imbalance or homeostasis loss^19^. Outward deflection (when opposite charge needles become farther than predicted) could reduce the treatment effectiveness by lowering the electroporated volume, inducing non-electroporated regions, and increasing tumor recurrence risks^9^. Because the effects of EP in animal tumor tissue are not directly observable, their outcome could take days or weeks (i.e*.*, there is no visual feedback at the time of the intervention)^20,21^, it is common to study this effects using both in vitro experiments with vegetal tissue and in silico computational models.

Vegetal models are used to assess EP volume and equipment testing as they show visual feedback through enzymatic browning when electroporated^22,23^. The ethics committee on the use of animals does not recommend in vivo animal testing for some scenarios involving suffering and pain, particularly for failure testing and effects analysis^24^. Tissue-mimicking phantoms such as potato tubers (*Solanum tuberosum*) are alternatives under the 3Rs (replacement, reduction and refinement) practice^24^. Also, computational models can calculate field distribution, optimal electrode positioning and geometries, and other relevant treatment parameters such as maximum demanded electric current, an equipment design requirement. A study may require that a comprehensive simulation of electrode positioning and geometries be performed to cover a possible ECT scenario. Nevertheless, computational modeling requires attention because each tissue may have a specific nonlinear electrical conductivity σ(E) model, where E is the local electric field^25^. The EP provokes an increase in the permeability of cells, which changes the electrical conductivity of the tissues^26,27^.

In this work, we performed in vitro and in silico experiments to investigate the interference of needle deflection in the electric field distribution and the risky limits of mechanical deformation for treatment success. By comparing the in vitro and in silico results, we evaluated whether the computational model can describe needle deflection cases. Additionally, we used an in silico tumor case model to demonstrate the effects that electrode deflections could impose in clinical scenarios. The simulation-based design may support recommendations on mechanical deformation tolerances. We evaluate the electric field distribution and maximum electric current, which are technical parameters or requirements used in ECT equipment design.

## Results

We observed ECT unsafe parameters during needle deflections. When performing +2 mm outward deflections, we show a potentially dangerous electric field indentation. When performing −3 mm inward deflections, we show a device-damaging electric current. Moreover, the findings indicate that the *in silico* model may not describe the *in vivo* experiment.

**Figure 2:**
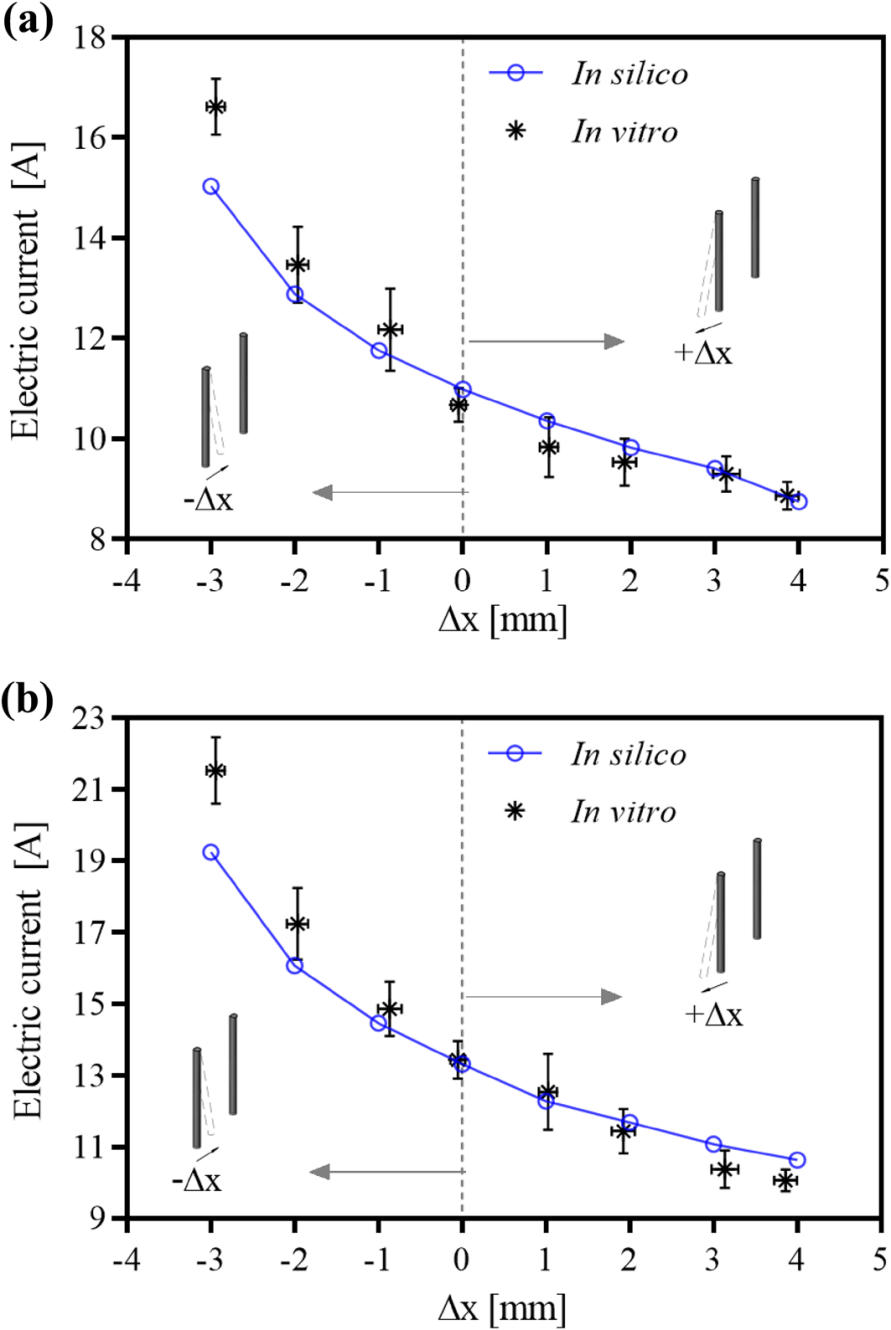
In silico and in vitro (mean and CI = 0.95) results of electric currents for (**a**) 3-pair and (**b**) 4-pair electrodes in *Solanum tuberosum* tissue. − Δx and + Δx represent inward and outward needle electrode deflections, respectively. The Δx uncertainty measurements are presented as means and CI = 0.95 (horizontal bars). In the case of 3 pairs, there are no statistical differences between the in silico and in vitro electric currents for Δx from −2 to 4 mm, and Δx from −1 to 2 mm in the case of 4 pairs.

**Figure 3:**
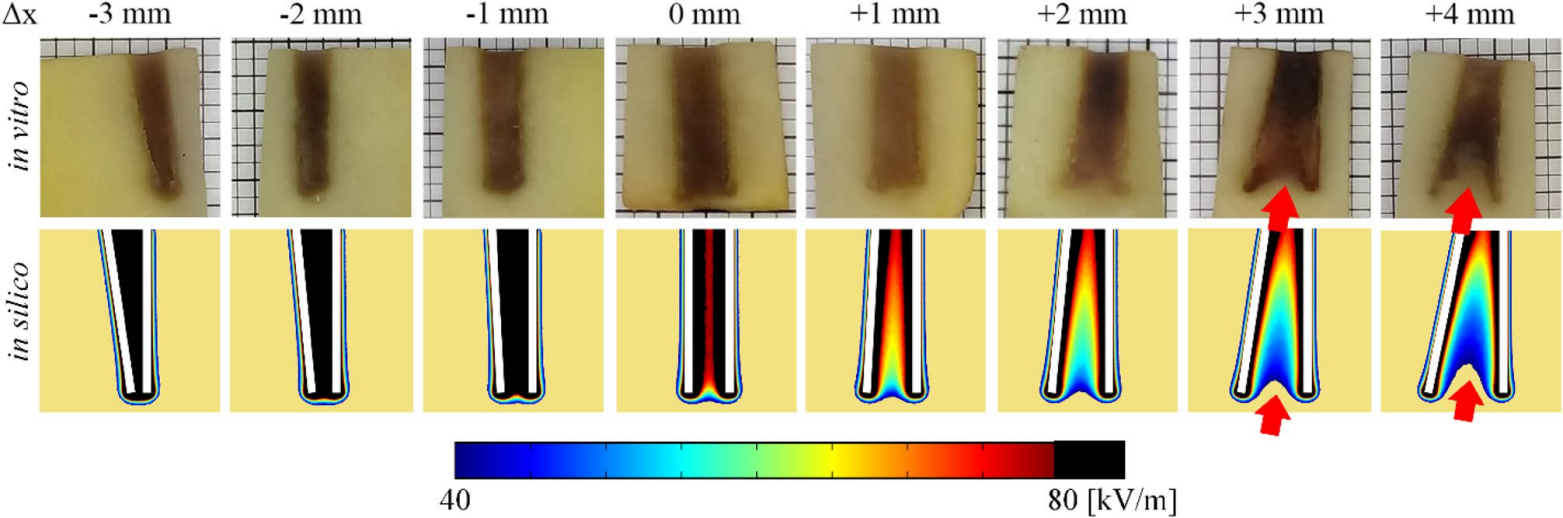
In vitro and in silico results of electric field distribution in *Solanum tuberosum* tissue. In vitro stained areas indicate tissue electroporation. The indentation effect (non-electroporated volume between the electrode pairs) can be observed from Δx =  + 3 mm. The cut plane is shown in Fig. 1f highlight. The in silico cut planes were generated by COMSOL Multiphysics v.5.1 (COMSOL Inc., Stockholm, Sweden; https://www.comsol.com/comsol-multiphysics).

Figure 2 shows the in silico and in vitro (mean and CI) values for each needle deflection groups for the *Solanum tuberosum* tissue. Figure 2 data is given by Supplementary Tables S1 to S4 (electric currents samples, mean and CI, relative error, and p-value) and Supplementary Table S6 (Δx samples of uncertainty measurements, means and CI). In silico relative error is less than 12%. However, there are cases with statistical differences (i.e., 3 pair Δx = −3 mm and 4-pair Δx = −3, −2, 3 and 4). The 4-pair model diverges more than the 3-pair (when comparing differences among deflection steps). Up to 55% and 60% in vitro electric current increase was observed for 3-pair and 4-pair electrodes, respectively. The current change is not linearly dependent on the deflection degree (see the in silico solid blue line in Fig. 2), which is a consequence of the complex field distribution due to the geometry. Figure 3 shows the electric field distribution for both in silico and in vitro experiments using the 3-pair electrode. The stained area on in silico results indicates electric field intensity above 40 kV/m, which is the typical ERE threshold for potato tissue^22,28–30^. The darkly stained region on in vitro results represents electroporated tissue. We observed stains starting from approximately 40 kV/m. The electric field indentation was observed in vitro and in silico (see the red arrows in Fig. 3). The called indentation, also known as electric field blind spot, is a region without an adequate electric field for electroporation, thus, may cause treatment failure. Similar results were obtained in the 4-pair electrode samples.

**Figure 4:**
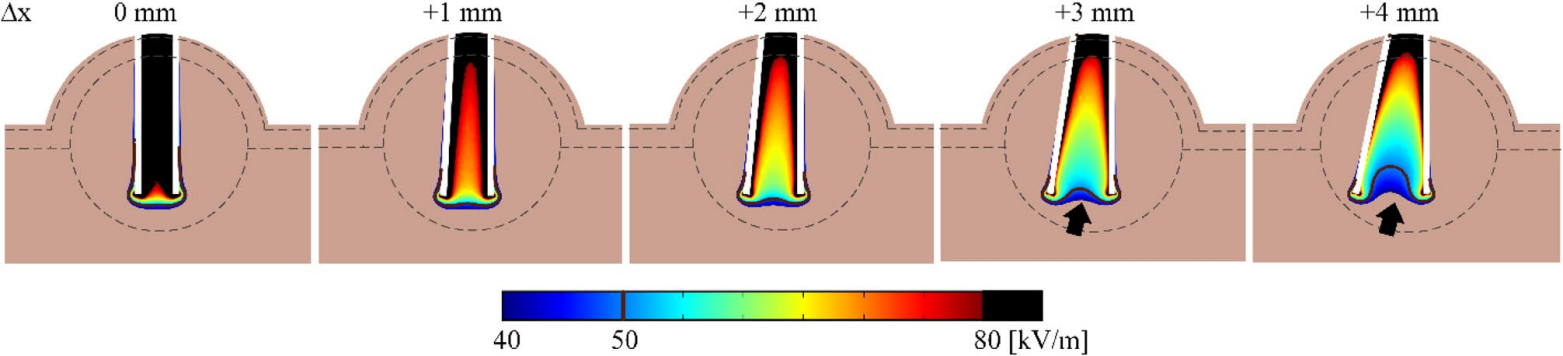
Electric field distribution in tumor tissue case study in + Δx. In the range of 50 kV/m, the indentation (non-electroporated volume between the electrode pairs) was observed from Δx =  + 3 mm. The electric field of 50 kV/m is a typical reversible electroporation threshold. The cut planes were generated by COMSOL Multiphysics v.5.1 (COMSOL Inc., Stockholm, Sweden; https://www.comsol.com/comsol-multiphysics).

**Figure 5:**
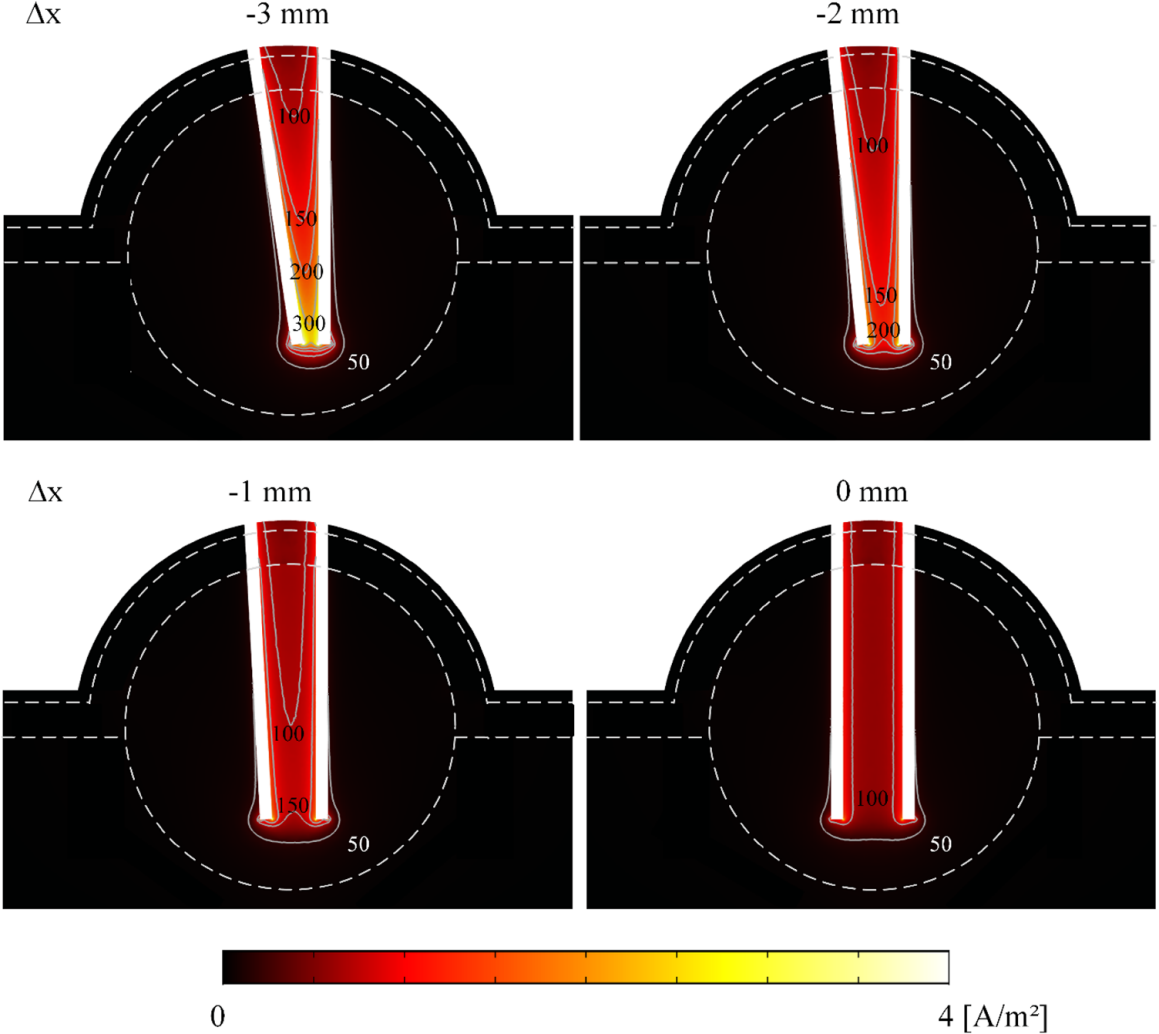
Electric current density distribution and electric field (kV/m) lines with inward deflection in tumor tissue case study. Electric field intensities higher than 150 kV/m are observed in a large tissue area for Δx smaller than −1 mm. The cut planes were generated by COMSOL Multiphysics v.5.1 (COMSOL Inc., Stockholm, Sweden; https://www.comsol.com/comsol-multiphysics).

Figures 4 and 5 show the tumor case study results using the 3-pair electrode. Figure 4 depicts the electric field distribution for + Δx cases and the indentation effect. The tumor case study has indentation similar to the vegetal study (see the arrows in Figs. 3 and 4). Figure 5 shows the electric current density for −Δx cases; the charge concentration can lead to fourfold current density increase at the point of the needles (up to 4·10E5 A/m^2^). We observed that when using the 4-pair electrode, the total electrical currents are 30.6 A, 25.0 A, 21.5 A and 18.7 A for −3 mm, 2 mm, 1 mm, and 0 mm deflection, respectively (for 3-pair case check the Supplementary Table S5). The tumor tissue electric current is 50–60% higher than the vegetal tissue; this is expected due to the higher electrical conductivity.

## Discussion

Electroporation requires a certain level of local electric field. Therefore, one of the ECT pillars is the electric field distribution. The needle electrodes are reused in veterinary ECT. Some of them can be damaged during use, leading to deflection that attracts attention from an electrical point of view. The immediate threat is the change in the electric field distribution. Specifically, the inward deflection can cause the equipment to shut down, and outward deflection can cause blind spots in the area between the electrodes that is normally used as a volumetric treatment reference. The currently EP tissue modeling also may need further adjustment to be compatible with higher field strength. In vitro and in silico results suggest that ±2 mm may be the tolerance limit for mechanical damage. Higher values may lead to electrode failure.

There are reports of reuse of needle electrode in veterinary ECT^10,11^. The electrodes may be mechanically stressed by autoclave sterilization or by multiple perforations. Excessive deflection by reuse may result in a non-predicted distribution of the electric field. Inward deflection may cause excessive current and undesirable thermal ablation, and outward deflection may provoke indentation in the electric field and a reduced electroporated tissue volume. Caution must be taken by the practitioners to avoid a treatment failure. The effects of EP cannot be directly observed during treatment^20,21^, and it is important to perform equipment failure tests and effect analyzes. We used a potato tuber as a tool to validate in silico electrode deflection models (through electric current measurements, data shown in Fig. 2) and as visual feedback of the electric field distribution (see the in vitro potato tuber stains in Fig. 3).

The electric field distribution (see Fig. 3) shows that inward deflection reduces the electroporated area in the outer regions. Outward deflection induces indentation (non-electroporated volumes in the region between needles, see the indicated by the red arrows in Fig. 3). These electric field distortions must be avoided, as non-treated regions are associated with clinical tumor recurrence^9^. In our study, the vegetable tissue was used as EP visual feedback. However, in animal or clinical ECT, treatment feedback may take days or weeks (*i.e.*, cancer reduction or tissue necrosis). Figure 4 shows that in the tumor tissue case study, there are also non-electroporated areas between the outward deflected needles, similarly to vegetal outcomes. If the electrode has a deflection, the volume in between the base of the needles should not be used as a surgical reference. Inward deflection of less than -2 mm requires higher electric current limits from the EP hardware. Many commercial electroporators have a maximum delivery current of about 20 A^31,32^. Exceeding the equipment limits may cause the hardware shutdown or damage, provoking a treatment failure.

The statistical analyzes reveal that the vegetal in silico model proposed by Ivorra et al*.*^22^ is adequate for an electrode deflection of ± 1 mm (4-pair electrode) and ± 2 mm (3-pair electrode). *Solanum tuberosum* has already been validated as in vitro electroporation electric fields model-dependent for plates and needle electrodes^26,29^. However, when the deflection overpasses a certain level, the in silico experiments do not describe the in vitro experiments. The mismatch could be explained by the fact that the model was built to best fit the ESOPE recommendations (40 to 120 kV/m). However, severe deflections can induce a wide range of electric field amplitude into the tissue. In the inward deflection, especially for the 4-pair electrode variant (see Fig. 2), we can also observe that the in vitro experiments lead to higher electric currents than the simulations (also observed for the 3-pair configuration in Fig. 2). If the in silico model does not describe the in vitro worst-case scenario, it may lead to incorrect equipment specifications. Thus, caution is required when simulating electric field in supra-ESOPE, a discussion we have not found in the current literature. If the simulation cannot predict an electrical overcurrent, the user will face a possible hardware shutdown. Besides, in silico model is designed to describe the effects of tissue conductivity regarding electroporation only. However, in real scenarios, other phenomena can act secondarily in increasing currents in the tissue when it is submitted to electric fields that surpass the irreversible threshold (Δx between −1 and −3 mm). Reports from literature show a temperature rising in the electrodes and surrounding tissues when submitted to the standard ECT protocol^33^, and that both cell permeabilization and Joule heating collaborate to electrical conductivity increase due to EP^34^. It is possible that Joule effect played a role in the extrapolated electric current (Fig. 2) and current density (Fig. 5) we observed in the cases of higher needle proximity. We aim to conduct further studies on describing the conductivity changes in electrical conductivity during EP.

The model deviation could also explain the problems faced by other authors when building new electrode arrangements with complex electric field distribution^35^. ESOPE electrode variations have regular shapes with a minimally disordered electric field distribution, as we can see in Figs. 3, 4 and 5 with no needle deflection (Δx = 0). However, newly developed electrodes design could give rise to regions of higher or lower electric field intensity, which consequently operate outside of the model. Further studies are needed to evaluate new EP tissue models.

Needle insertion is associated with tissue deformation that may exceed the mechanical resistance of the material. Needle tip geometry also influences deflection. Previous work has shown that the single beveled tip needle is not suitable for procedures that require precision^16,36^. In addition, other studies have shown that needles with canonical and multi beveled tips have less deflection than the single beveled ones^37,38^. There is no reported standardization for the chamfer used in needle electrodes, including ESOPE's. The pointy ends of the needles can assume different proportions on different manufacturers, which could generate a slightly different electric field distribution among them. However, the results generated from our choice of bending resistant needles, yet non-beveled, still unveil and alert the restrictions bound to an effective ECT treatment facing needle deflections.

We recommend avoiding ESOPE Type II with more than 2 mm of inward or outward deflection electrodes. Based on our findings, these effects can be harmful to the patient and equipment and should be considered irreversible damage or tumor recurrence, and the electrodes should be replaced in these situations. We encourage that other ECT equipment damage and its effects be discussed in future research. The ECT involves the application of low-frequency and pulsed electric current, which is sensitive to the electrodes assemble. If electrical parts are displaced from their original position, this may pose a safety risk. We recommend ECT practitioners to be aware of possible needle deflections before and after each insertion into the tissue to avoid the combination of deflections caused by various artifacts that could lead to unsuccessful tumor treatment.

## Conclusion

We investigated the failure mode of electrode deflection in the ESOPE electrodes type II and its consequences. The deflection changes the electric field distribution, which depends mainly on EP. The inward deflection increases the electric current through the tissue and it may not be described by current ECT conductivity models. The outward deflection could induce non-electroporated volume in internal regions, possibly provoking treatment failure and tumor recurrence if the practitioner is not aware of the risks involving electrode deflections. The use of a damaged electrode is not recommended. However, a tolerance of ± 2 mm may be adequate if deformation occurs during electrode insertion. Deformation to a greater extent than ± 2 mm may be risky to the success of the electrochemotherapy.

## Methods

We performed both in silico and in vitro experiments to evaluate the electric field distribution and electric current from different arrangements of ESOPE Type II needle electrode^9^ deflection. The radial direction of a deflected needle randomly occurs when it is inserted in tissues. However, we considered two directions (Δx): inward (Δx < 0) and outward (Δx > 0) deflection. In those cases, the anode needles were deflected, whereas the cathode ones were kept fixed, as shown in Fig. 1f. We considered that all needle pairs were deflected.

Electrode dimensions follow the ESOPE standards with 3 and 4 parallel pairs of needles. Each pair consists of two needles with 1 mm diameter, 20 mm height, and separated by 4 mm on inner edges in its standard configuration (no deflection, Δx = 0). In both 3 and 4 pairs variations, the inner edges of identically charged needles are 3 mm apart. All needles were fully inserted into the biological tissue (20 mm insertion). EP protocol also follows ESOPE recommendations with eight square-wave electric pulses of 400 V (100 kV/m electric field is expected as needles inner edges are at a 4 mm separation gap), 100 µs of pulse duration and 1 Hz of repetition rate. Because the current EP equipment do not adjust the voltage, we do not realize any voltage compensation due to electrode deflection.

### In silico experiments

The computational model was performed by the finite element method (FEM) software COMSOL Multiphysics v.5.1 (COMSOL Inc., Stockholm, Sweden). The mathematical model that describes the electric field distribution in biological systems can be modelled by the Laplace equation in the steady-state regime, as shown in Eq. (1).

1 $$-\nabla \cdot \left(\sigma \cdot \nabla V\right)=0$$

where σ is the electrical conductivity of the biological tissue dependent on the electric field (S/m), and V is the applied electric potential (V). The boundary conditions were all insulating on the external surfaces (Neumann’s boundary condition), and the Dirichlet’s boundary condition modelled the tissue-electrode contact. Equation (2) shows the sigmoid function used by Ivorra et al.^22^ to describe potato tissue conductivity changes through EP.

2 $$\sigma \left(\left|E\right|\right)=0.03+0.35\cdot {e}^{{-e}^{-0.01\cdot (\left|E\right|-250)}}$$

where σ is the potato tissue electrical conductivity (S/m), and E the local electric field.

The geometric models were built using COMSOL Geometric Tools. A cylinder with 60 mm diameter and 40 mm height was used to represent potato tissue. The electrode was modelled with the already reported dimensions. The mesh was generated with COMSOL Mesh Creation Tool at the ‘Finer’ resolution, resulting in approximately three million tetrahedral elements for each geometric problem. A total of eight simulations (from Δx = −3 mm to Δx = 4 mm, 1 mm step) were performed for each standard electrode and deflections cases. Calculations were run on a cluster server (Intel Xeon Gold 6126 @ 2.60 GHz, 20 cores, 300 GB RAM) with Ubuntu Linux (× 64, Canonical Ltd., London, United Kingdom) operating system.

**Figure 6:**
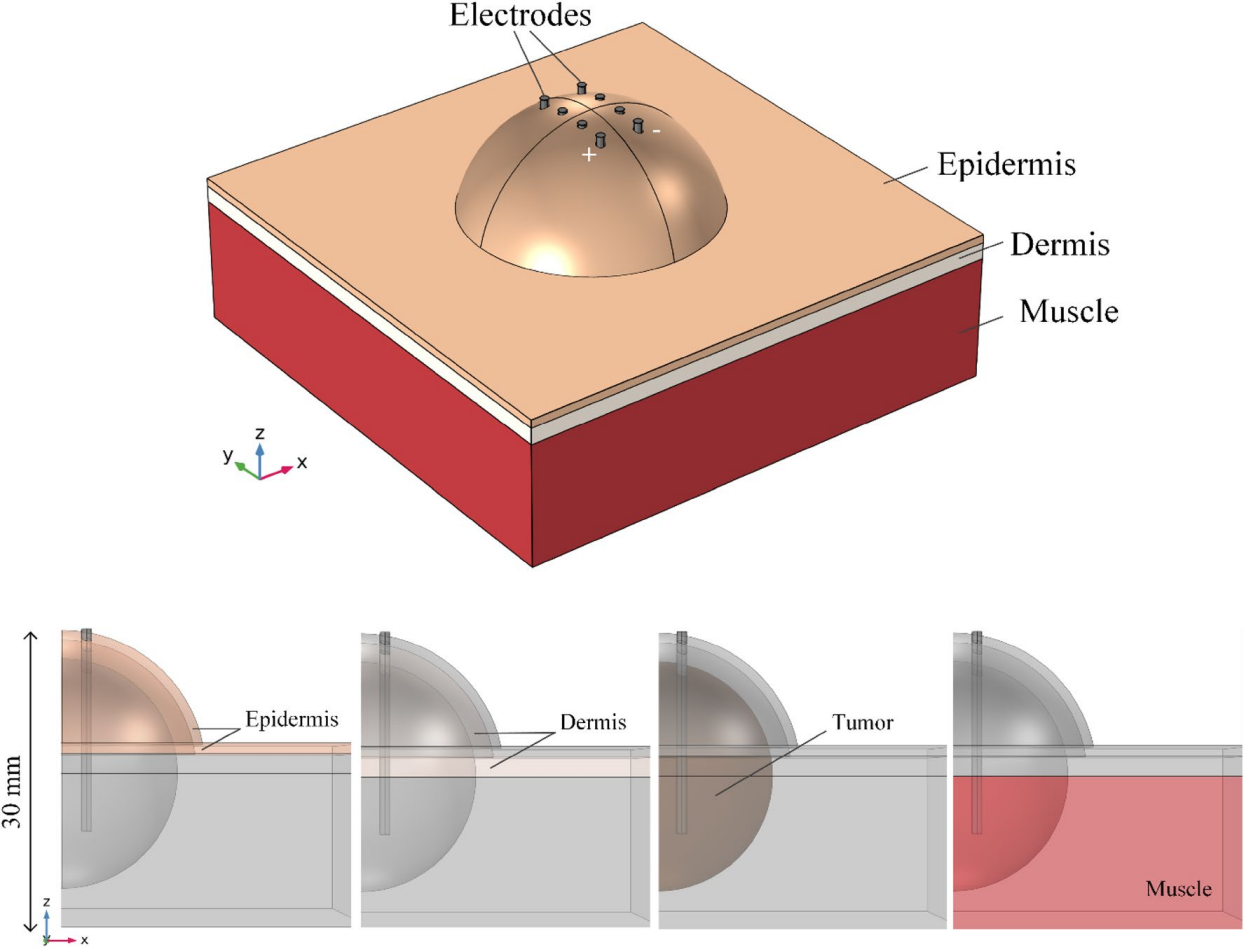
Numerical geometry of the tumor case study with ESOPE Type II electrode in its standard configuration. The case consists of a subcutaneous tumor. The tumor mass growth forces the upper tissues to spherically reshape. All geometry planes were generated by COMSOL Multiphysics v.5.1 (COMSOL Inc., Stockholm, Sweden; https://www.comsol.com/comsol-multiphysics).

Real scenarios of ECT involve treating tumors with irregular shapes and surrounded by other tissues or organs^5,39^. Therefore, we modeled the geometry of a subcutaneous tumor neighboring the epidermis and dermis tissues to mimic such an environment. Equation (3) shows the EP model described by Miklavičič et al.^40^.

3 $$\sigma \left(E\right)={\sigma }_{0}+\frac{{\sigma }_{MAX}-{\sigma }_{0}}{1+D\cdot {e}^{-\left(\frac{E-A}{B}\right)}}$$

$$A=\frac{{E}_{IRE}+{E}_{RE}}{2}\quad B=\frac{{E}_{IRE}-{E}_{RE}}{C}$$

where E_RE_ and E_IRE_ are the electric field thresholds of reversible and irreversible EP, σ_0_ and σ_max_ are the initial and maximum tissue conductivity, respectively. C = 8 and D = 10 are model constants. Table 1 shows the electrical parameters of tissues used in the in silico case. Figure 6 shows the in silico tumor case. The geometry consists of epidermis, dermis, and muscle blocks with respective thicknesses of 1 mm, 2 mm, and 25 mm. The width and depth of all tissues are 60 mm. Tumor tissue is a sphere of 12 mm diameter that projects under dermis and epidermis tissues. The projections of dermis and epidermis tissues are 14 mm and 15 mm, respectively. Their respective thicknesses are kept. The total height of the geometry is 30 mm. The mesh generated approximately three million six hundred thousand tetrahedral elements for each deflection geometry.

**Table 1:** Electrical parameters of tissues used in the tumor case simulations^25,43^.

Tissue	${\sigma }_{0}$ (S/m)	${\sigma }_{MAX}$ (S/m)	${E}_{RE}$ (kV/m)	${E}_{IRE}$ (kV/m)
Epidermis	0.008	0.8	40	120
Dermis	0.25	1	30	120
Tumor	0.3	0.75	40	80
Muscle	0.135	0.34	20	80

### In vitro experiments

Potato tubers (*Solanum tuberosum*) were purchased from local stores. The producer (Rio Bonito Orgânicos, Itatinga, Brazil) is certified by the Brazilian Ministry of Agriculture, Livestock and Food Supply (MAPA) audit for organically grown products. The Organic Conformity Assessment Organization accredited by MAPA provides the traceability of the product at https://conecta.paripassu.com.br/ (traceability code PPGHO390M0FIHIHB). This study complies with relevant institutional, national, and international guidelines and legislation on experimental research and field studies on plants or plant material.

The vegetables were not peeled and received a minimum number of cuts, followed by drying the electroporated surfaces before and after the pulse application to minimize artefacts unrelated to the EP process^30^. Eight groups of electrode deflection (from Δx = −3 mm to Δx = 4 mm, 1 mm step) per number of pairs (3 and 4) were performed with ten samples per group. We developed an external apparatus to guarantee electrode insertion with respective deflections. The apparatus can be seen in Supplementary Fig. S1a. The electrode consisted of independent parts of the anode and cathode needle sets for 3 and 4 pairs of Stainless Steel 316L (Ortobio LTDA, BR, ANVISA 80062900008). This material offers suitable bending stiffness^41^, thus, minimizing misalignment effects when inserted in potato tissue. Along with the electrodes, we designed external spacers. Spacers are 20 mm in height and consist of two fiberglass boards with 1.1 mm diameter orifices perforated on each board.

The electrode parts were placed in the tissue through orifices of spacers. For all bottom boards, the orifices distances mimicked ESOPE type II electrodes, as in real scenarios, the configuration is maintained at the base of the electrode. To ensure the desired Δx, we shifted the perforation places of anode orifices at each upper board spacer (1 mm step), as shown in Supplementary Fig. S1b. For a + 3 mm Δx, for example, anode needles were inserted into the upper board with a 1 mm distance from the cathode ones. We shortened the height of the + 4 mm Δx spacer to 10 mm. In this case, the anode row perforations distanced 2 mm of the cathode row (check Supplementary Fig. S1c). It ensured proper positioning and electrical safety.

To deliver a consistent analysis of the in vitro distribution of electric fields, we also performed Δx measurements on another set of samples (nonelectroporated ones), eight for each Δx configuration. After insertion into tubers, we cut slices precisely at each needle pair insertion and measured each Δx using an analogic caliper Mitutoyo 530–312 (Mitutoyo Corporation, Kanagawa, Japan). Finally, we calculated each average and 95% confidence interval (Supplementary Table S6).

Electric pulse was applied to the main samples using a programmable custom pulse generator^42^. The samples were kept in Petri dishes for 24 h at 25 ºC. Lastly, we cut slices perpendicularly to the electroporated surface, similar to the slices of in vitro Δx tests, as shown in Fig. 1f highlight. The slices were photographed under a lighting control chamber, with a 13 MP, f. 2.2 LG M250F digital camera (LG, South Korea).

The electric current of each application was measured using a digital oscilloscope Tektronix DPO2012B (Tektronix, USA) and electric current probe Tektronix A622 (Tektronix, USA). We registered the average electric current of the last pulse protocol. The average current was measured in the top of the square wave and disregarding the firsts and lasts 10 µs from the pulse edges to ensure that there are no transient effects.

### Data analysis and statistics

In our data set, we need to know how the in silico electrical current samples compare to our population (the corresponding in vitro results). We have 16 situations to test. For each situation, there is an in vitro group and an in silico sample. They are based on the Δx (−3, −2, −1, 0, 1, 2, 3 and 4) and the number of electrode pairs (3 and 4). We first tested whether the in vitro groups were approximately normally distributed using Shapiro–Wilk normality test. The data must be normally distributed to test means. We found that all in vitro groups were approximately normally distributed. No data transformation was performed to adjust for normality. We used a one-sample t-test to test the probability that the in vitro mean was equal to the in silico value. The t-test was chosen because of the small sample size (10 samples). Variance in the in vitro electric current data is expected due to the heterogeneity of the tissue. We found no tolerable error consent in the ECT area. We included a 95% confidence interval (CI = 0.95) and a 5% significance level (α = 0.05) in all statistical calculations. In silico and in vitro were also compared from another perspective, namely the relative error, i.e*.*, the percentage difference between the means. We note that comparing multiple groups is not appropriate (i.e*.*, comparing two levels of displacement) because they are electrically different situations that should not be compared.

We also applied the Shapiro–Wilk normality test to the in vitro measurements of Δx uncertainty. Again, all groups presented normal distribution, and the results were expressed by averages. All data analyses were performed using the RStudio statistical software package (RStudio v. 1.2, Inc, 2019).

## Supplementary Information

Supplementary Information.

## Data Availability

All data generated or analyzed during this study are included in this published article (and its Supplementary Information files).
