# Supplementary material for: Electrochemotherapy treatment safety under parallel needle deflection
Source: Sci Rep. 2022 Feb 17;12:2766. doi: 10.1038/s41598-022-06747-x (PMC8854592; doi:10.1038/s41598-022-06747-x)
Supplement: Supplementary file 1 — Supplementary Information. [file 41598_2022_6747_MOESM1_ESM.pdf]

Electrochemotherapy Treatment Safety Under Parallel Needle Deflection

Daniella L. L. S. Andrade[1], Raul Guedert[1], Guilherme B. Pintarelli[1], Marcelo M. M. Rangel[2], Krishna D. Oliveira[2], Priscila G. Quadros[2] & Daniela O. H. Suzuki[1,*]

[1]*Institute of Biomedical Engineering, Federal University of Santa Catarina, Florianópolis, 88040-900, Brazil*

[2]*Oncology Veterinary, VetCâncer, São Paulo, 04523-013, Brazil*

[*] Daniela O. H. Suzuki; daniela@ppgeel.ufsc.br

SUPPLEMENTARY INFORMATION

Supplementary **Table S1**: *In vitro* electric currents of *Solanum tuberosum* for the 3-pair electrode. Ten samples were tested for each Δx. Each value represents the average current of the last (eighth) pulse of the EP protocol.

Δx [mm]	*In vitro* samples [A]									
	n1	n2	n3	n4	n5	n6	n7	n8	n9	n10
-3	16.2	17.4	15.8	17.4	15.68	15.4	16.7	17.2	17	17.4
-2	14.6	13.8	13.2	12.4	14.4	12.48	13.4	12.4	12.6	15.4
-1	10.8	11.4	12.6	11.4	12.4	10.72	11.64	13.6	13.8	13.4
0	10.6	10.2	11	10.2	11.2	9.92	10.88	10.6	10.8	11.4
1	9.2	9	11.2	9.6	10.2	9.4	10.2	9.4	11.2	9
2	9.8	9.2	9.4	9.4	10.2	8.6	9.4	11	9.2	9.2
3	9.2	9.6	8.6	8.8	9.8	9.4	10	9.8	9	8.8
4	8.6	8.8	8.6	9.2	9	9.2	8.2	9.4	9.2	8.5

Supplementary **Table S2**: *In silico* and *in vitro* (mean and C.I. 95%) results of the electric currents for the 3-pair electrode in *Solanum tuberosum*. Δx = 0 represents no deflected needles. Δx from -2 to 4 show no statistical difference between *in silico* and *in vitro* analysis of electric currents.

Δx [mm]	*In silico* [A]	*In vitro* (mean and CI) [A]	Relative Error	p-value
-3	15.04	16.62 (16.06 - 17.18)	10.51%	<0.0001
-2	12.89	13.47 (12.71 - 14.23)	5.23%	0.104
-1	11.76	12.18 (11.35 - 13.00)	3.55%	0.2676
0	10.99	10.68 (10.34 - 11.02)	-2.81%	0.0527
1	10.36	9.84 (9.24 - 10.43)	-5.05%	0.0621
2	9.83	9.54 (9.07 - 10.01)	-2.95%	0.1824
3	9.41	9.30 (8.95 - 9.65)	-1.14%	0.4989
4	8.75	8.87 (8.59 - 9.14)	1.35%	0.3421

Supplementary **Table S3**: *In vitro* electric currents of *Solanum tuberosum* for the 4-pair electrode. Ten samples were tested for each Δx. Each value represents the average current of the last (eighth) pulse of the EP protocol.

Δx [mm]	*In vitro* samples [A]									
	n1	n2	n3	n4	n5	n6	n7	n8	n9	n10
-3	21.8	20	19.2	22.6	21.6	22	21.2	20.8	23.6	22.6
-2	16.4	17.2	18.2	17.6	16.4	15.2	19.6	15.6	17.2	19
-1	15.6	14.8	15	15.6	15.6	14.4	12.8	13.6	16.4	14.8
0	12.8	14.2	13.8	14.8	12.4	12.6	13.4	13.8	13.2	13.4
1	13.4	14.4	13.2	14.4	12.6	10.6	10.4	10.8	12.6	13
2	11.6	11.8	11.2	11.4	11	12.8	11.6	12	11.6	9.4
3	10.2	10.4	10.6	10.8	10.6	10.6	9.8	11	11.2	8.6
4	10.4	10.6	9.52	10	9.4	10.4	10.4	9.8	9.8	10.4

Supplementary **Table S4**: *In silico* and *in vitro* (mean and C.I. 95%) results of the electric currents for the 4-pair electrode in *Solanum tuberosum*. Δx = 0 represents no deflected needles. Δx from -1 to 2 show no statistical difference between *in silico* and *in vitro* analysis of electric currents.

Δx [mm]	*In silico* [A]	*In vitro* (mean and CI) [A]	Relative Error	p-value
-3	19.25	21.54 (20.61 - 22.47)	11.88%	<0.0001
-2	16.07	17.24 (16.23 - 18.25)	7.27%	0.0176
-1	14.46	14.86 (14.10 - 15.62)	2.73%	0.2533
0	13.32	13.44 (12.91 - 13.97)	0.92%	0.6061
1	12.29	12.54 (11.48 - 13.60)	1.96%	0.6126
2	11.69	11.44 (10.82 - 12.06)	-2.19%	0.3635
3	11.08	10.38 (9.851 - 10.91)	-6.33%	0.0076
4	10.64	10.07 (9.769 - 10.38)	-5.38%	0.0005

Supplementary **Table S5**: *In silico* electric currents of tumor tissue with needle deflection (Δx) for the 3-pair electrode.

Δx [mm]	*In silico* [A]
-3	23.9
-2	19.7
-1	17.0
0	14.6

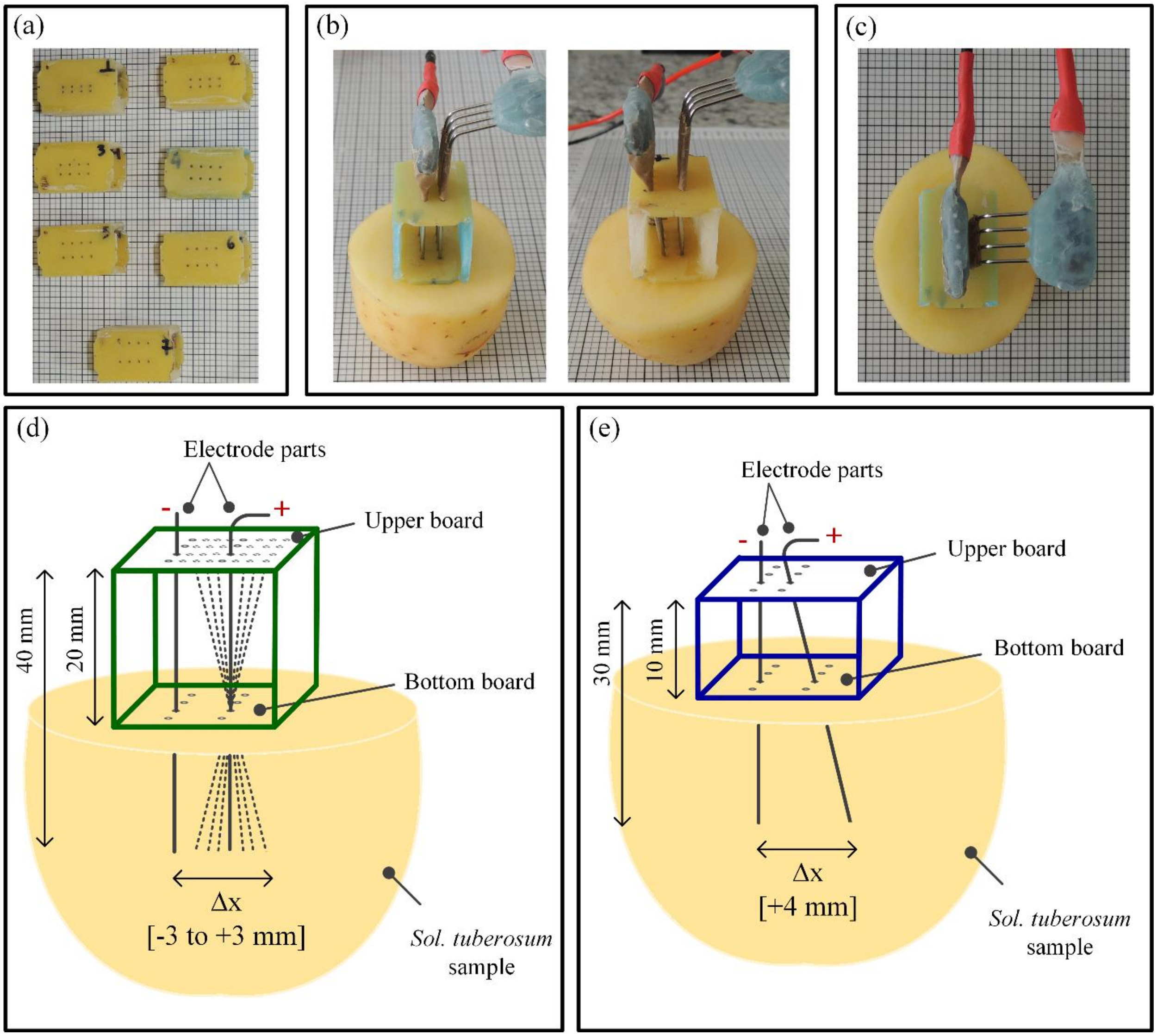

Supplementary **Figure S1**: External apparatus used to produce respective Δx in *Solanum tuberosum*. (a) shows the external spacers, (b) and (c) show the electrode parts (4 pairs) inserted into samples, Δx = 0 mm and Δx = - 3 mm. (d) and (e) show the scheme designed to produce needle tip distances. The electrode parts are inserted into samples through the perforations placed in the bottom and upper boards. Illustrations of the scheme were created in Microsoft VISIO Professional 2019 v.2111 (Microsoft Corporation, Washington, USA; https://www.microsoft.com/en-us/microsoft-365/visio/flowchart-software).

Supplementary **Table S6**: Uncertainty measurements of Δx (mean and C.I. 95%).

Δx [mm]	Measured Δx [mm]								Δx means and CI [mm]	p-value
	n1	n2	n3	n4	n5	n6	n7	n8		
-3	-2.8	-3.1	-3.1	-3.0	-2.8	-2.8	-3.0	-2.9	-2.94 (-3.05; -2.83)	0.0881
-2	-2.1	-1.9	-1.9	-2.0	-2.0	-1.7	-1.9	-2.2	-1.96 (-2.10; -1.84)	0.741
-1	-0.8	-1.0	-1.0	-1.1	-0.6	-0.9	-0.7	-0.8	-0.86 (-1.00; -0.72)	0.8619
0	-0.1	-0.1	-0.2	-0.1	0.1	-0.1	0.1	0.0	-0.05 (-0.14; 0.04)	0.1199
1	1.2	1.1	0.9	1.0	0.8	1.1	1.1	1.0	1.02 (0.92; 1.13)	0.592
2	2.1	1.7	2.0	2.0	1.7	2.0	1.8	2.1	1.92 (1.78; 2.06)	0.0672
3	3.2	3.3	3.5	3.1	3.0	3.0	2.9	3.1	3.14 (2.98; 3.30)	0.6025
4	3.7	3.8	3.7	3.7	4.0	4.0	3.9	4.1	3.86 (3.73; 4.00)	0.1623
